# Measuring inter-rater reliability for nominal data – which coefficients and confidence intervals are appropriate?

**DOI:** 10.1186/s12874-016-0200-9

**Published:** 2016-08-05

**Authors:** Antonia Zapf, Stefanie Castell, Lars Morawietz, André Karch

**Affiliations:** 1 Department of Medical Statistics, University Medical Center Göttingen, Humboldtallee 32, 37073 Göttingen, Germany; 2 Department of Epidemiology, Helmholtz Centre for Infection Research, Inhoffenstrasse 7, 38124 Braunschweig, Germany; 3 Institute of Pathology, Diagnostik Ernst von Bergmann GmbH, Charlottenstr. 72, 14467 Potsdam, Germany; 4 ESME - Research Group Epidemiological and Statistical Methods, Helmholtz Centre for Infection Research, Inhoffenstrasse 7, 38124 Braunschweig, Germany; 5 German Center for Infection Research, Hannover-Braunschweig site, Göttingen, Germany

**Keywords:** Inter-rater heterogeneity, Fleiss’ kappa, Fleiss’ K, Krippendorff’s alpha, Bootstrap, Confidence interval

## Abstract

**Background:**

Reliability of measurements is a prerequisite of medical research. For nominal data, Fleiss’ kappa (in the following labelled as Fleiss’ K) and Krippendorff’s alpha provide the highest flexibility of the available reliability measures with respect to number of raters and categories. Our aim was to investigate which measures and which confidence intervals provide the best statistical properties for the assessment of inter-rater reliability in different situations.

**Methods:**

We performed a large simulation study to investigate the precision of the estimates for Fleiss’ K and Krippendorff’s alpha and to determine the empirical coverage probability of the corresponding confidence intervals (asymptotic for Fleiss’ K and bootstrap for both measures). Furthermore, we compared measures and confidence intervals in a real world case study.

**Results:**

Point estimates of Fleiss’ K and Krippendorff’s alpha did not differ from each other in all scenarios. In the case of missing data (completely at random), Krippendorff’s alpha provided stable estimates, while the complete case analysis approach for Fleiss’ K led to biased estimates. For shifted null hypotheses, the coverage probability of the asymptotic confidence interval for Fleiss’ K was low, while the bootstrap confidence intervals for both measures provided a coverage probability close to the theoretical one.

**Conclusions:**

Fleiss’ K and Krippendorff’s alpha with bootstrap confidence intervals are equally suitable for the analysis of reliability of complete nominal data. The asymptotic confidence interval for Fleiss’ K should not be used. In the case of missing data or data or higher than nominal order, Krippendorff’s alpha is recommended. Together with this article, we provide an R-script for calculating Fleiss’ K and Krippendorff’s alpha and their corresponding bootstrap confidence intervals.

**Electronic supplementary material:**

The online version of this article (doi:10.1186/s12874-016-0200-9) contains supplementary material, which is available to authorized users.

## Background

In interventional as well as in observational studies, high validity and reliability of measurements are crucial for providing meaningful and trustable results. While validity is defined by how well the study captures the measure of interest, high reliability means that a measurement is reproducible over time, in different settings and by different raters. This includes both the agreement among different raters (inter-rater reliability, see Gwet [1]) as well as the agreement of repeated measurements performed by the same rater (intra-rater reliability). The importance of reliable data for epidemiological studies has been discussed in the literature (see for example Michels et al. [2] or Roger et al. [3]).

The prerequisite of being able to ensure reliability is, however, the application of appropriate statistical measures. In epidemiological studies, information on disease or risk factor status is often collected in a nominal way. For nominal data, the easiest approach for assessing reliability would be to simply calculate observed agreement. The problem of this approach is that “this measure is biased in favour of dimensions with small number of categories” (Scott [4]). In order to avoid this problem, two other measures of reliability, Scott’s pi [4] and Cohen’s kappa [5], were proposed, where the observed agreement is corrected for the agreement expected by chance. As the original kappa coefficient (as well as Scott’s pi) is limited to the special case of two raters, it has been modified and extended by several researchers so that various formats of data can be handled [6]. Although there are limitations of kappa, which have already been discussed in the literature (e.g., [7–9]), kappa and its variations are still widely applied. A frequently used kappa-like coefficient was proposed by Fleiss [10] and allows including two or more raters and two or more categories. Although the coefficient is a generalization of Scott’s pi, not of Cohen’s kappa (see for example [1] or [11]), it is mostly called Fleiss’ kappa. As we do not want to perpetuate this misconception, we will label it in the following as Fleiss’ K as suggested by Siegel and Castellan [11].

An alternative measure for inter-rater agreement is the so-called alpha-coefficient, which was developed by Krippendorff [12]. Alpha has the advantage of high flexibility regarding the measurement scale and the number of raters, and, unlike Fleiss’ K, can also handle missing values.

Guidelines for reporting of observational studies, randomized trials and diagnostic accuracy studies [13–15] request that confidence intervals should always be provided together with point estimates as the meaning of point estimates alone is limited. For reliability measures, the confidence interval defines a range in which the true coefficient lies with a given probability. Therefore, a confidence interval can be used for hypothesis testing. If, for example, the aim is to show reliability better than chance at a confidence level of 95 %, the lower limit of the two-sided 95 % confidence interval has to be above 0. In contrast, if a substantial reliability is to be proven (Landis and Koch [16] define *substantial* as a reliability coefficient larger than 0.6, see below), the lower limit has to be above 0.6. For Fleiss’ K, a parametric asymptotic confidence interval (CI) exists, which is based on the delta method and on the asymptotic normal distribution [17, 18]. This confidence interval is in the following referred to as “asymptotic confidence interval”. An alternative approach for the calculation of the confidence intervals for K is the use of resampling methods, in particular bootstrapping. For the special case of two categories and two raters, Klar et al. [19] performed a simulation study and recommended using bootstrap confidence intervals when assessing the uncertainty of kappa (including Fleiss’ K). For Krippendorff’s alpha, bootstrapping offers the only suitable approach, because the distribution of alpha is unknown.

The assessment of reliability in epidemiological studies is heterogeneous, and often uncertainty is not taken into account, which results in an inappropriate methodological use. Moreover, there is a lack of evidence which reliability measure performs best under different circumstances (with respect to missing data, prevalence distribution and number of raters or categories). Except for a study by Häußler [20], who compared measures of agreement for the special case of two raters and binary measurements, there is no systematic comparison of reliability measures available. Therefore, it was our aim to

- compare Fleiss’ K and Krippendorff’s alpha (as the most generalized measures for agreement in the framework of inter-rater reliability) regarding the precision of their estimates;
- compare the asymptotic CI for Fleiss’ K with the bootstrap CIs for Fleiss’ K and Krippendorff’s alpha regarding their empirical coverage probability;
- give recommendations on the measure of agreement and confidence interval for specific settings.

## Methods

Fleiss’ K is based on the concept that the observed agreement is corrected for the agreement expected by chance. Krippendorff’s alpha in contrast is based on the observed disagreement corrected for disagreement expected by chance. This leads to a range of −1 to 1 for both measures, where 1 indicates perfect agreement, 0 indicates no agreement beyond chance and negative values indicate inverse agreement. Landis and Koch [16] provided cut-off values for Cohen’s kappa from poor to almost perfect agreement, which could be transferred to Fleiss’ K and Krippendorff’s alpha. However, e.g., Thompson and Walter [7] demonstrated that reliability estimates strongly depend on the prevalence of the categories of the item investigated. Thus, interpretation based on simple generalized cut-offs should be treated with caution, and comparison of values across studies might not be possible.

### Fleiss’ K

From the available kappa and kappa-like coefficients we chose Fleiss’ K [10] for this study because of its high flexibility. It can be used for two or more categories and two or more raters. However, similarly to other kappa and kappa-like coefficients, it cannot handle missing data except by excluding all observations with missing values. This implies that all *N* observations are assessed by *n* raters, and that all observations with less than *n* ratings are deleted from the dataset. For assessing the uncertainty of Fleiss’ K, we used the corrected variance formula by Fleiss et al. [18]. The formulas for the estimation of Fleiss’ K, referred to as $\widehat{\mathrm{K}}$, and its standard error $se\left(\widehat{\mathrm{K}}\right)$ are given in the Additional file 1 (for details see also Fleiss et al. [21], pages 598–626). According to Fleiss [18], this standard error is only appropriate for testing the hypothesis that the underlying value is zero. Applying the multivariate central limit theorem of Rao [22], an approximate normal distribution can be assumed for large samples under the hypothesis of randomness [18]. This leads to the asymptotic two-sided 1 – α confidence interval

$$ C{I}_{asymp}\left(\mathrm{K}\right)=\left[\widehat{\mathrm{K}}\pm {z}_{1-\alpha /2}\ se\left(\widehat{\mathrm{K}}\right)\right] $$

with *z*_1 − *α*/2_ as quantile of the standard normal distribution. The asymptotic CI is by definition generally only applicable for large sample sizes; moreover, Efron [23] stated that the delta method in general tends to underestimate the standard error, leading to too narrow confidence intervals and to an inflation of the type-one error. Therefore, several authors proposed resampling methods [24–26] as an alternative for calculating confidence intervals for Fleiss’ K. We will here use a standard bootstrap approach, as suggested by Klar et al. [19] and Vanbelle et al. [26]. In each bootstrap step *b* = 1, …, *B* a random sample of size N is drawn with replacement from the N observations. Each observation drawn contains the associated assessments of all raters. For each bootstrap sample the point estimate is calculated, denoted by ${\widehat{\mathrm{K}}}_b$. The vector of the point estimates, sorted by size, is given by ${\mathbf{K}}_B=\left({\widehat{\mathrm{K}}}_{\left[1\right]},\dots,\ {\widehat{\mathrm{K}}}_{\left[B\right]}\right)$. Then the two-sided bootstrap confidence interval for the type-one error α is defined by the empirical α/2 and 1 – α/2 percentiles of **K**_*B*_:

$$ C{I}_{Bootstrap}\left(\mathrm{K}\right)=\left[{\widehat{\mathrm{K}}}_{\left[B\cdot \alpha /2\right]};{\widehat{\mathrm{K}}}_{\left[B\cdot \left(1-\alpha /2\right)\right]}\right]\ . $$

### Krippendorff’s alpha

Krippendorff [12] proposed a measure of agreement, which is even more flexible than Fleiss’ K, called Krippendorff’s alpha. It can also be used for two or more raters and categories, and it is not only applicable for nominal data, but for any measurement scale, including metric data. Another important advantage of Krippendorff’s alpha is that it can handle missing values, given that each observation is assessed by at least two raters. Observations with only one assessment have to be excluded.

The formulas for the estimation of Krippendorff’s alpha Â are given in the Additional file 1. For details, we refer to Krippendorff’s work [27]. Gwet [1] points out that Krippendorff’s alpha is similar to Fleiss’ K, especially if there are no missing values. The difference between the two measures is explained by different definitions of the expected agreement. For the calculation of the expected agreement for Fleiss’ K, the sample size is taken as infinite, while for Krippendorff’s alpha the actual sample size is used.

For Krippendorff’s alpha the theoretical distribution is not known, even not an asymptotic one [28]. However, the empirical distribution can be obtained by the bootstrap approach. Krippendorff proposed an algorithm for bootstrapping [28, 29], which is also implemented in the SAS- and SPSS-macro from Hayes [28, 30]. The proposed algorithm differs from the one described for Fleiss’ K above regarding three aspects. First, the algorithm weights for the number of ratings per individual to account for missing values. Second, not the N observations, with each observation containing the associated assessments of all raters, are randomly sampled. Instead the random sample is drawn from the coincidence matrix, which is needed for the estimation of Krippendorff’s alpha (see Additional file 1). This means that the dependencies between the raters are not taken into account. The third difference is that Krippendorff keeps the expected disagreement fixed, and only the observed disagreement is calculated anew in each bootstrap step. We performed simulations for a sample size of *N* = 100 observations, which showed that the empirical and the theoretical coverage probability differ considerably (median empirical coverage probability of 60 %). Therefore, we decided to use in our study the same bootstrap algorithm for Krippendorff’s alpha as for Fleiss’ K (in the following labelled as standard approach). This leads to a vector of the bootstrap estimates (sorted by size) **Α**_*B*_ = (Â_[1]_, …, Â_[*B*]_). Then the bootstrap 1 – α/2 confidence interval is defined by the percentiles:

$$ C{I}_{Bootstrap}\left(\mathrm{A}\right)=\left[{\widehat{\mathrm{A}}}_{\left[B\cdot \alpha /2\right]};{\widehat{\mathrm{A}}}_{\left[B\cdot \left(1-\alpha /2\right)\right]}\right]\ . $$

### R-script K_alpha

As there is no standard software, where Fleiss’ K and Krippendorff’s alpha with bootstrap confidence intervals are implemented (for an overview see Additional file 2), we provide an R-script together with this article, named “K_alpha”. The R-function kripp.alpha from the package irr [31] and the SAS-macro kalpha from Andrew Hayes [30] served as reference. The function K_alpha calculates Fleiss’ K (for nominal data) with the asymptotic and the bootstrap interval and Krippendorff’s alpha with the standard bootstrap interval. The description of the program as well as the program itself, the function call for a fictitious dataset and the corresponding output are given in the Additional file 3.

### Simulation study

We performed a simulation study in R 3.2.0. The simulation program can be obtained from the authors. In the simulation study, we investigated the influence of four factors:

- the number of observations, i.e., *N = 50, 100, 200*
- the number of raters, i.e., *n = 3, 5, 10*
- the number of categories, i.e., *k = 2, 3, 5.*
- the strength of agreement (low, moderate and high), represented by Fleiss’ K and Krippendorff’s alpha ∈ *[0.4,0.93]* (see below)

This resulted in a total of 81 scenarios. The choice of factor levels was motivated by the real world case study used in this article and by scenarios found frequently in the literature.

**Fig. 1:**
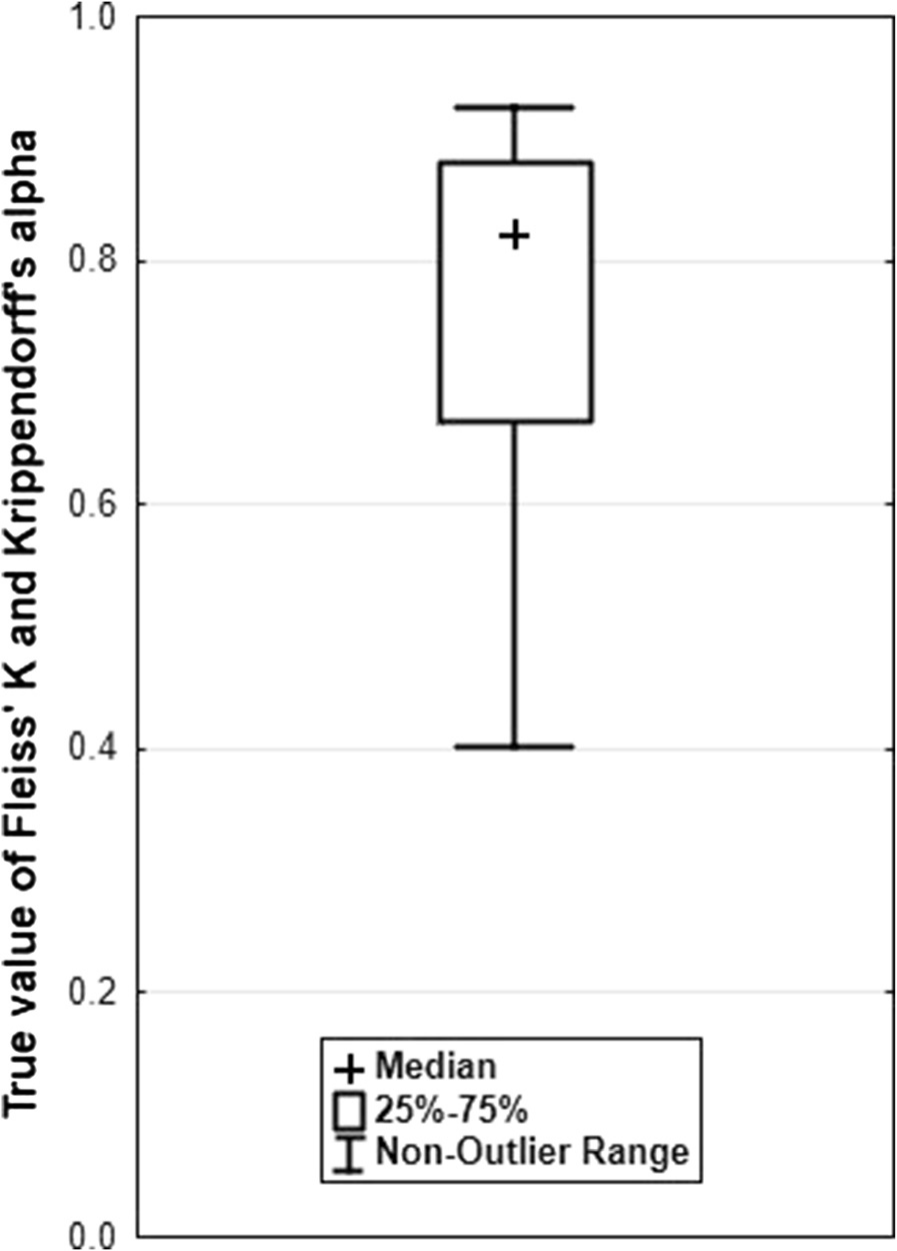
Distribution of the true values in the 27 scenarios (independent of the sample size)

We generated nominal data by using the multinomial distribution with *N* subjects, *n* raters, and *k* categories because Fleiss’ K in its unweighted version is only appropriate for nominal data. Varying the probabilities of the multinomial distribution between 0.1 and 0.5 led to true parameters between 0.40 and 0.93 on the [−1; 1] scale; in half of the scenarios the true value lied between 0.67 and 0.88 (see Fig. 1). The true values for Krippendorff’s alpha and Fleiss’ K differed only at the fourth to fifth decimal place.

We used 1,000 simulation runs and 1,000 bootstrap samples for all scenarios in accordance with Efron [23], and set the two-sided type-one error to 5 %. For each simulated dataset, we calculated Fleiss’ K with the two-sided 95 % asymptotic and the bootstrap confidence interval, and Krippendorff’s alpha with the two-sided 95 % bootstrap interval. We investigated two statistical criteria: bias and coverage probability. The mean bias is defined by the mean point estimates over all simulation runs minus the true value given. The number of simulation runs, in which the true value was located inside the two-sided 95 % confidence interval divided by the total number of simulation runs, gives the empirical coverage probability.

For three specific scenarios, we deleted (completely at random) a pre-specified proportion of the data (10, 25, and 50 %) in order to evaluate the ramifications of missing values under the missing completely at random (MCAR) assumption. The selection criteria for the scenarios were an empirical coverage probability close to 95 % for Fleiss’ K *and* Krippendorff’s alpha, a sample size of 100, as well as variation in agreement, categories and raters over the scenarios.

The three scenarios, each for a sample size of 100, are:

- I: five raters, a scale with two categories and low agreement
- II: five raters, a scale with five categories and high agreement
- III: ten raters, a scale with three categories and medium agreement.

Then we applied the standard bootstrap algorithm for Fleiss’ K and Krippendorff’s alpha to investigate the robustness against missing values.

### Case study

In order to illustrate the theoretical considerations learnt from the simulation study, we applied the same approach to a real world dataset focusing on the inter-rater agreement in the histopathological assessment of breast cancer as used for epidemiological studies and clinical decision-making. The first *n* = 50 breast cancer biopsies of the year 2013 that had been sent in for routine histopathological diagnostics at the Institute of Pathology, Diagnostik Ernst von Bergmann GmbH (Potsdam, Germany), were retrospectively included in the study. For the present study, the samples were independently re-evaluated by four senior pathologists, who are experienced in breast cancer pathology and immunohistochemistry, and who were blinded to the primary diagnosis and immunohistochemical staining results. Detailed information is provided in the Additional file 4.

## Results

### Simulation study

**Fig. 2:**
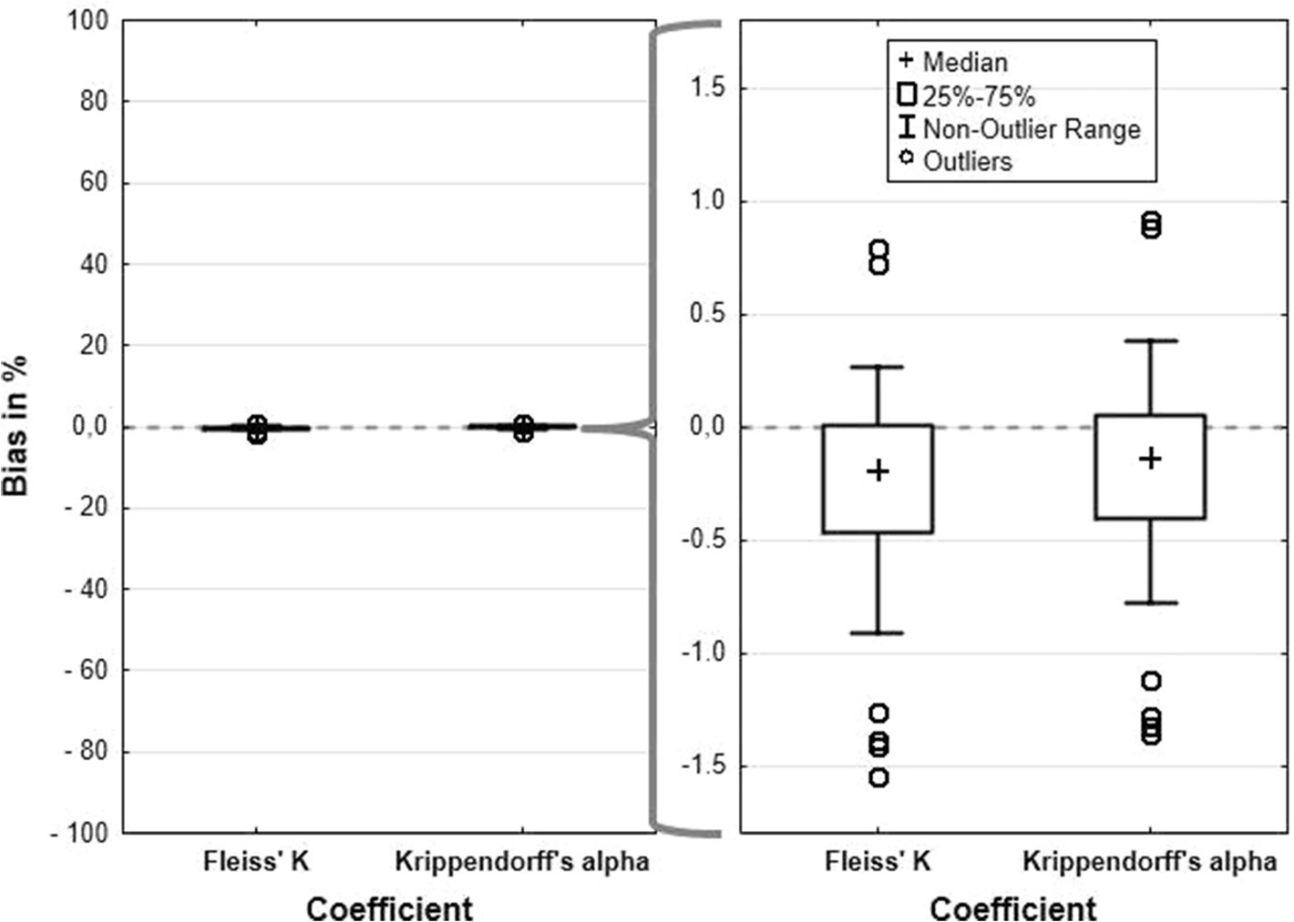
Percentage bias for Krippendorff’s alpha and Fleiss’ K over all 81 scenarios. The dotted line indicates unbiasedness. On the left side the whole range from −100 to +100 % is displayed, on the right side the relevant excerpt is enlarged

Point estimates of Fleiss’ K and Krippendorff’s alpha did neither differ considerably from each other (Additional file 5 above) nor from the true values over all scenarios (Fig. 2).

**Fig. 3:**
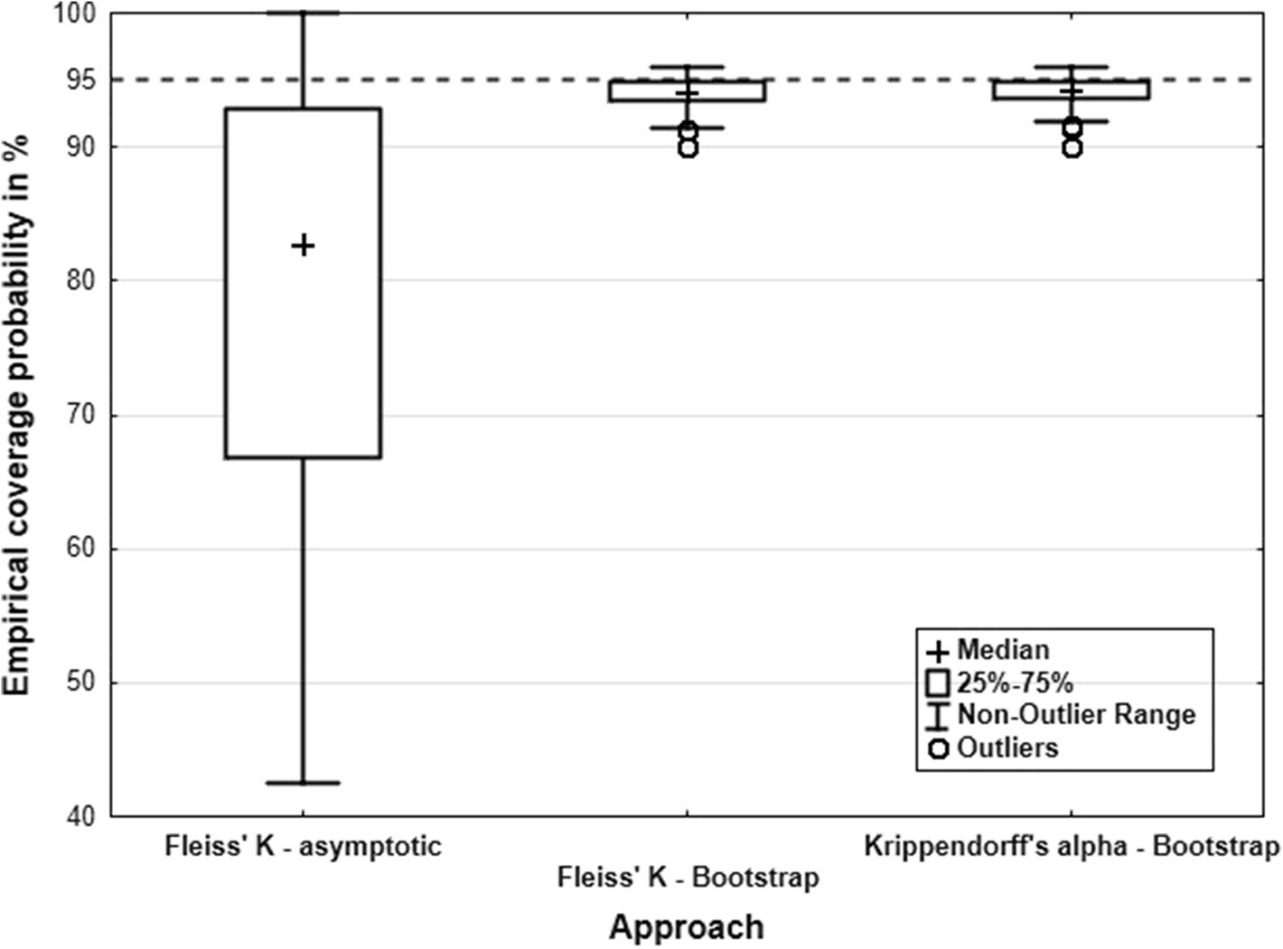
Two-sided empirical type-one error of the three approaches over all 81 scenarios. The dotted line indicates the theoretical coverage probability of 95 %

Regarding the empirical coverage probability, it could be shown that the asymptotic confidence interval for Fleiss’ K leads to a low coverage probability in most cases (and also for a sample size up to 1000, see Additional file 5 below), while the bootstrap intervals for Krippendorff’s alpha and Fleiss’ K provide virtually the same results and the empirical coverage probability is close to the theoretical one (Fig. 3).

**Fig. 4:**
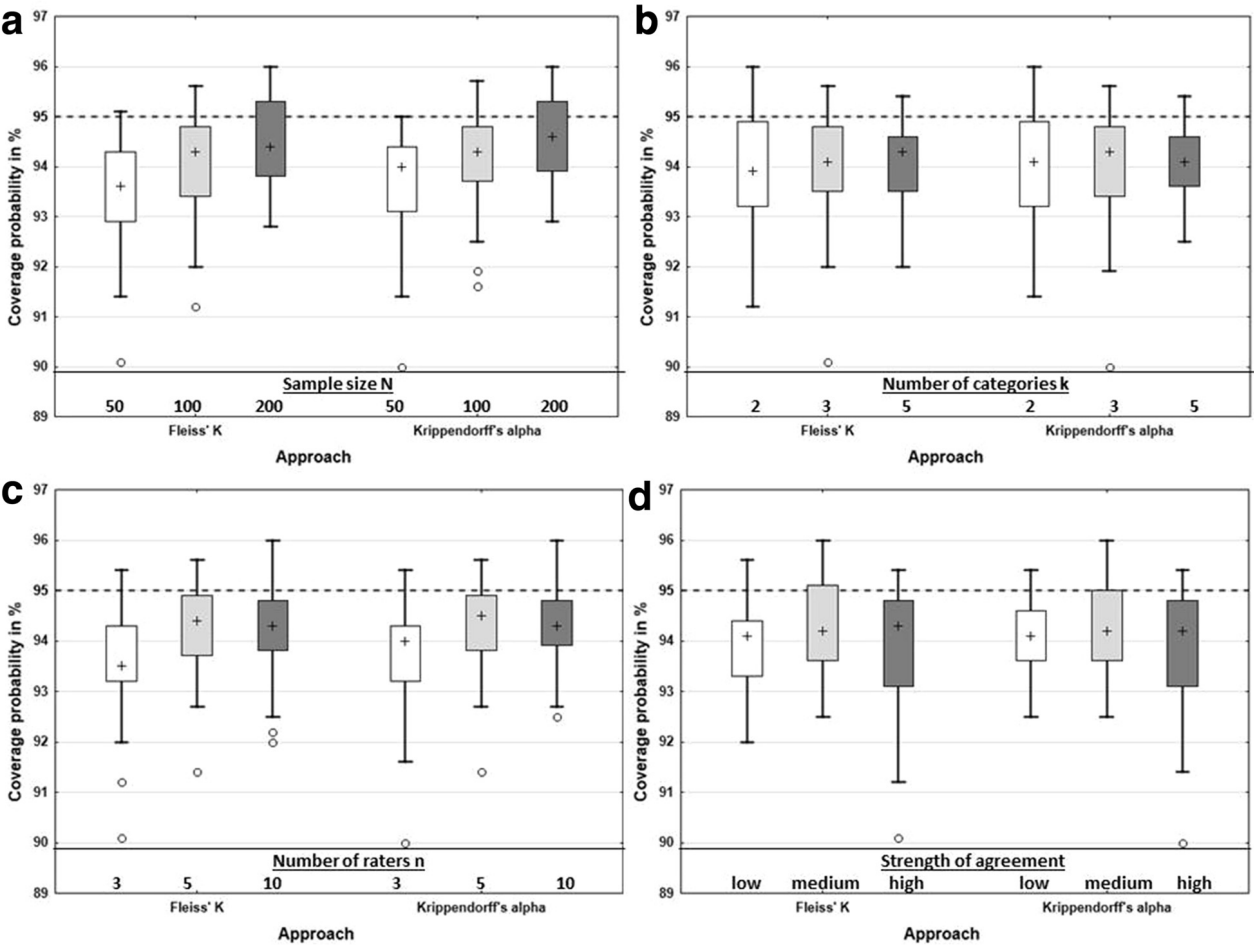
Empirical coverage probability for the bootstrap intervals for Krippendorff’s alpha and Fleiss’ K with varying factors sample size (**a**), number of categories (**b**), number of raters (**c**) and strength of agreement (**d**). In each subplot, summary results over all levels of the other factors are displayed. The dashed line indicates the theoretical coverage probability of 95 %

We investigated the effect of each variation factor individually; to do so, we fixed one factor at a given level, then varied the levels of all other factors, and reported results over these simulation runs. It can be seen that with larger sample sizes the median empirical coverage probability gets closer to the nominal level of 95 % for Krippendorff’s alpha as well as for Fleiss’ K (Fig. 4a). For a sample size of 200, the median empirical coverage probability is quite close to the theoretical of 95 %. With increasing number of categories the range of the coverage probability gets smaller (Fig. 4b). For three raters, the coverage probability is below that for five or ten raters, while for five and ten raters the coverage probability is more or less the same (Fig. 4c). With increasing strength of agreement, the median empirical coverage probability tends to get closer to 95 % for both coefficients (Fig. 4d).

Missing values cannot be considered by Fleiss’ K except by excluding all observations with missing values. In contrast, for Krippendorff’s alpha all observations with at least two assessments are included in the calculation. We investigated the robustness of both coefficients in the case of missing values under MCAR conditions with respect to the mean bias and the empirical two-sided type-one error for three scenarios (I. *N* = 100, *n* = 5, k = 2, low agreement; II. *N* = 100, *n* = 5, k = 5, high agreement; III. *N* = 100, *n* = 10, k = 3, medium agreement; see also methods section). Krippendorff’s alpha was very robust against missing values, even if 50 % of the values were missing. In contrast, Fleiss’ K was unbiased only in the case of 10 % missing values in all three scenarios. For 50 % missing values, in all three scenarios the bias was larger than 20 % and the coverage probability was below 50 % (Table 1).

**Table 1:** Empirical coverage probability and bias in % of Krippendorff’s alpha and Fleiss’ K for simulated data with varying percentage of missing values

	Missing values	Krippendorff’s alpha		Fleiss’ K	
		Coverage probability (%)	Bias (%)	Coverage probability (%)	Bias (%)
I	10 %	95.4	- 0.82	94.4	- 0.78
	25 %	94.3	- 0.54	94.3	- 1.40
	50 %	93.9	- 0.67	40.8	- 25.93
II	10 %	92.9	0.04	95.2	- 0.16
	25 %	94.7	0.03	67.7	8.27
	50 %	93.6	0.01	13.3	- 25.72
III	10 %	95.1	0.01	93.8	- 0.26
	25 %	95.2	−0.02	65.5	- 7.76
	50 %	94.8	−0.13	33.3	- 23.72

The scenarios are defined as: I. *N* = 100, *n* = 5, k = 2, low agreement; II. *N* = 100, *n* = 5, k = 5, high agreement; III. *N* = 100, *n* = 10, k = 3, medium agreement (with N as number of observations, n as number of raters and k as number of categories)

### Results of the case study

Observed agreement in the case study showed considerable differences between the parameters investigated (Table 2), ranging from 10 % (MIB-1 proliferation rate) to 96 % (estrogen receptor group). Parameters based on semi-objective counting (i.e., hormone receptor groups and MIB-1 proliferation) had no higher agreement than parameters based on pure estimation.

**Table 2:** Results of the case study (*n* = 50) of histopathological assessment of patients with mamma carcinoma rated by four independent and blinded readers. The six ordinal parameters were also assessed if as they were measured in a nominal way

Parameter	Levels	Scale	Missing values (in %)	Observed agreement	Fleiss’ K			Krippendorff’s alpha	
					Point estimate	Asymptotic CI	Bootstrap CI	Point estimate	Bootstrap CI
Estrogen IRS	2	Nominal	0	96 %	0.88	0.76–0.99	0.65–1.00	0.88	0.66–1.00
MIB-1 status	2	Nominal	0	72 %	0.66	0.55–0.78	0.51–0.80	0.66	0.51–0.80
HER-2 status	3	Nominal	0	86 %	0.77	0.68–0.87	0.58–0.90	0.77	0.60–0.92
Estrogen intensity	4	Nominal	0	78 %	0.62	0.54–0.71	0.42–0.78	0.62	0.40–0.79
		Ordinal			-	-	-	0.74	0.51–0.80
Estrogen group	5	Nominal	0	86 %	0.74	0.66–0.82	0.55–0.88	0.74	0.55–0.89
		Ordinal			-		-	0.88	0.73–0.96
Progesteron intensity	4	Nominal	10	77 %	0.74	0.63–0.84	0.56–0.89	0.69	0.53–0.83
		Ordinal			-	-	-	0.86	0.75–0.93
Progesteron group	5	Nominal	0	44 %	0.56	0.50–0.63	0.43–0.66	0.56	0.45–0.67
		Ordinal			-	-	-	0.83	0.72–0.90
HER-2 score	4	Nominal	0	46 %	0.52	0.45–0.60	0.38–0.64	0.52	0.37–0.65
		Ordinal			-	-	-	0.70	0.53–0.82
MIB-1 proliferation rate	10	Nominal	0	10 %	0.20	0.15–0.25	0.12–0.28	0.20	0.12–0.27
		Ordinal			-	-	-	0.81	0.68–0.87

With respect to the comparison of both measures of agreement, point estimates for all variables of interest did not differ considerably between Fleiss’ K and Krippendorff’s alpha irrespective of the observed agreement or the number of categories (Table 2). As suggested by our simulation study, confidence intervals were narrower for Fleiss’ K when using the asymptotic approach than when applying the bootstrap approach. The relative difference of both approaches became smaller the lower the observed agreement was. There was no relevant difference between the bootstrap confidence intervals for Fleiss’ K and Krippendorff’s alpha.

For the three measures used for clinical decision-making (MIB-1 state, HER-2 status, estrogen IRS), point estimates between 0.66 and 0.88 were observed, indicating some potential for improvement. Alpha and Fleiss K’ estimates for the six other measures (including four to ten categories) varied from 0.20 to 0.74.

In the case of missing data (progesteron intensity), Krippendorff’s alpha showed a slightly lower estimate than Fleiss’ K which is in line with the results of the simulation study.

For variables with more than two measurement levels, we also assessed how the use of an ordinal scale instead of a nominal one affected the predicted reliability. As Fleiss’ K does not provide the option of ordinal scaling, we performed this analysis for Krippendorff’s alpha only. Alpha estimates increased by 15–50 % when using an ordinal scale compared to a nominal one. However, use of an ordinal scale gives for these variable correct estimates of alpha as data were collected in an ordinal way. Here, we could obtain point estimates from 0.70 (HER-2 score) to 0.88 (estrogen group) indicating substantial agreement between raters.

## Discussion

We compared the performance of Fleiss’ K and Krippendorff’s alpha as measures of inter-rater reliability. Both coefficients are highly flexible as they can handle two or more raters and categories. In our simulation study as well as in a case study, point estimates of Fleiss’ K and Krippendorff’s alpha were very similar and were not associated with over- or underestimation. The asymptotic confidence interval for Fleiss’ K led to a very low coverage probability, while the standard bootstrap interval led to very similar and valid results for both, Fleiss’ K and Krippendorff’s alpha. The limitations of the asymptotic confidence interval approach are linked to the fact that the underlying asymptotic normal distribution holds only true for the hypothesis that the true Fleiss’ K is equal to zero. For shifted null hypotheses (we simulated true values between 0.4 and 0.93), the standard error is no longer appropriate [18, 23]. As bootstrap confidence intervals are not based on assumptions about the underlying distribution, they offer a better approach in cases where the derivation of the correct standard error for specific hypotheses is not straight forward [24–26].

In a technical sense, our conclusions are only valid for the investigated simulation scenarios, which we, however, varied in a very wide and general way. Although we did not specifically investigate if the results of this study can be transferred to the assessment of intra-rater agreement, we are confident that the results of our study are also valid for this application area of Krippendorff’s alpha and Fleiss’ K as there is no systematic difference in the way the parameters are assessed. Moreover, the simulation results for the missing data analysis are only valid for MCAR conditions as we did not investigate scenarios in which data were missing at random or missing not at random. However, in many real-life reliability studies the MCAR assumption may hold as missingness is indeed completely random, for example because each subject is only assessed by a random subset of raters due to time, ethical or technical constraints.

Interestingly, Krippendorff’s alpha is, compared to the kappa coefficients (including Fleiss’ K), rarely applied in practice, at least in the context of epidemiological studies and clinical trials. A literature search performed in Medline, using the search terms Krippendorff’s alpha or kappa in combination with agreement or reliability (each in title or abstract), led to 11,207 matches for kappa and only 35 matches for Krippendorff’s alpha from 2010 up to 2016 (2016/03/01). When extracting articles published in the five general epidemiological journals with the highest impact factors (International Journal of Epidemiology, Journal of Clinical Epidemiology, European Journal of Epidemiology, Epidemiology, and American Journal of Epidemiology) from the above described literature search, one third of the reviewed articles didn’t provide corresponding confidence intervals (18 of 52 articles which reported kappa or alpha values). Only in two of the reviewed articles with CIs for kappa, it was specified that bootstrap confidence intervals were used [32, 33]. In all other articles it was not reported if an asymptotic or a bootstrap CI was calculated. As bootstrap confidence intervals are not implemented in standard statistical packages, it must be assumed that asymptotic confidence intervals were used, although sample sizes were in some studies as low as 10 to 50 subjects [34, 35]. As our literature search was restricted to articles, in which kappa or Krippendorff’s alpha was mentioned in the abstract, there is the opportunity of selection bias. It can be assumed that in articles, which report reliability coefficients in the main text but not in the abstract, confidence intervals are used even less. This could also have influenced the observed difference in usage of kappa and Krippendorff’s alpha; however, is this case we do not think that the proportion of the two measures would be different.

In general, agreement measures are often criticized for the so-called paradoxa associated with them (see [9]). For example, high agreement rates might be associated with low measures of reliability, if the prevalence of one category is low. Krippendorff extensively discussed these paradoxa and identified them as conceptual problems in the understanding of observed and expected agreement [36]. We did not simulate such scenarios with unequal frequencies of categories or discrepant frequencies of scores between raters. However, as the paradoxa concern both coefficients likewise, because only the used sample size for the expected agreement differs (actual versus infinite), it can be assumed that there is no difference between alpha and Fleiss’ K in their behaviour in those situations.

An alternative approach to the use of agreement coefficients in the assessment of reliability would be to model the association pattern among the observers’ ratings. There are three groups of models which can be used for this: latent class models, simple quasi-symmetric agreement models, and mixture models (e.g.,) [37, 38]. However, this modelling approaches request a higher level of statistical expertise so that for standard applicants it is in general much simpler to estimate the agreement coefficients and especially to interpret them.

## Conclusion

In the case of nominal data and no missing values, Fleiss’ K and Krippendorff’s alpha can be recommended equally for the assessment of inter-rater reliability. As the asymptotic confidence interval for Fleiss’ K has a very low coverage probability, only standard bootstrap confidence intervals as used in our study can be recommended. If the measurement scale is not nominal and/or missing values (completely at random) are present, only Krippendorff’s alpha is appropriate. The correct choice of measurement scale of categorical variables is crucial for an unbiased assessment of reliability. Analysing variables in a nominal setting which have been collected in an ordinal way underestimates the true reliability of the measurement considerably, as can be seen in our case study. For those interested in a one-fits-all approach, Krippendorff’s alpha might, thus, become the measure of choice. Since our recommendations cannot easily be applied within available software solutions, we offer a free R-script with this article which allows calculating Fleiss’ K as well as Krippendorff’s alpha with the proposed bootstrap confidence intervals (Additional file 3).

## Abbreviations

CI, confidence interval; MCAR, missing completely at random; se, standard error

## Additional files

Additional file 1: Estimators - Estimators of Fleiss’ K (with standard error) and Krippendorff’s alpha. (DOCX 19 kb) Additional file 2: Available software – implementation of Fleiss’ K and/or Krippendorff’s alpha in most common statistical software programs used in epidemiology/biometry. (DOCX 14 kb) Additional file 3: R-script k_alpha – syntax, explanation, and analysis of a fictitious data set. (DOCX 25 kb) Additional file 4: Case study – description, dataset, syntax, and output. (DOCX 21 kb) Additional file 5: Figures A1 and A2 – scatter plot of the point estimates of Fleiss’ K versus Krippendorff’s alpha and empirical coverage probability of the asymptotic confidence interval for Fleiss’ K. (DOCX 92 kb)
