# Supplementary material for: Measuring inter-rater reliability for nominal data – which coefficients and confidence intervals are appropriate?
Source: BMC Med Res Methodol. 2016 Aug 5;16:93. doi: 10.1186/s12874-016-0200-9 (PMC4974794; doi:10.1186/s12874-016-0200-9)
Supplement: Additional file 1: — Estimators - Estimators of Fleiss’ K (with standard error) and Krippendorff’s alpha. (DOCX 19 kb) [file 12874_2016_200_MOESM1_ESM.docx]

**Additional file 1**

**Estimators of Fleiss' K (with standard error) and Krippendorff's alpha**

*Estimation of Fleiss’ K and its standard error*

The total number of subjects is denoted by *N*, the number of ratings per subject by *n*, and the number of categories by *k*. The index for the subject is *i* = 1,…,*N* and the index for the category is *j* = 1,…,*k*. Then $n_{ij}$ is the number of raters who classed the subject *i* into the category *j*. The proportion of all assignments to category *j* is defined as

$$p_{j}=\frac{1}{Nn} \sum_{i=1}^{N} n_{ij}$$

with $\sum_{j=1}^{k} n_{ij}=n_{i}=n$ (because of no missing values) and $\sum_{j=1}^{k} p_{j}=1$. Then the estimated K for category *j* is defined as

$$\hat{K}_{j}=1-\frac{\sum_{i=1}^{N} n_{ij}(n-n_{ij})}{Nn(n-1)p_{j}q_{j}}$$

with $q_{j}=1-p_{j}$ . This leads to an estimate of the overall K as

$$\hat{K}=\frac{\sum_{j=1}^{k} p_{j}q_{j}\hat{K}_{j}}{\sum_{j=1}^{k} p_{j}q_{j}}$$

Then the large sample standard error, derived by Fleiss et al. [1], which is appropriate for testing that the true value is 0, is

$$se\left( \hat{K} \right)=\frac{\sqrt{2}}{\sum_{j=1}^{k} p_{j}q_{j}\sqrt{Nn(n-1)}}\sqrt{\left( \sum_{j=1}^{k} p_{j}q_{j} \right)^{2}-\sum_{j=1}^{k} p_{j}q_{j}\left( {q_{j}-p}_{j} \right)} .$$

For details we refer also to Fleiss et al. [2], pages 598-626.

*Estimation of Krippendorff’s alpha*

As mentioned in the text, we assume that all *N* subjects were assessed by at least two raters. Then Krippendorff’s alpha in its most general form (for all measurement scales and two or more raters and categories) is defined by

$$A=1-\frac{D_{o}}{D_{e}}=\frac{\sum_{j} \sum_{j^{'}} o_{jj^{'}}\delta_{jj^{'}}^{2}}{\sum_{j} \sum_{j^{'}} e_{jj^{'}}\delta_{jj^{'}}^{2}} (\mathrm{category} j,j^{'}=1\ldots,k),$$

with $D_{o}$ as observed disagreement and $D_{e}$ as disagreement expected by chance [3]. The observed coincidences are defined as

$$o_{jj'}=\sum_{i=1}^{N} \frac{number of j-j^{'}pairs in subject i}{n_{i}-1},$$

where $n_{i}$ is the number of raters who assessed subject *i*. In contrast, the coincidences expected by chance are defined as

$$e_{jj'}=\left\{ \begin{aligned} {n_{j}(n_{j^{'}}-1)}/\left( \sum_{i} n_{i}-1 \right) \mathrm{if} j=j' \\ {n_{j}n_{j^{'}}}/\left( \sum_{i} n_{i}-1 \right) \mathrm{if} j\neq j' \end{aligned} \right.,$$

with $n_{j},n_{j'}$ as number of ratings in category $j, j^{'}=1,\ldots,k$. The metric-specific difference function $\delta_{jj'}^{2}$ for the categories $j,j^{'}=1,\ldots,k$ is defined as follows:

- Nominal scale: $\delta_{jj'}^{2}=\left\{ \begin{matrix} 0 \mathrm{if}j=j' \\ 1 \mathrm{if}j\neq j' \end{matrix} \right.$
- Ordinal scale: $\delta_{jj'}^{2}=\left( \frac{n_{j}}{2}+\sum_{g>j}^{g<j'} n_{g}+\frac{n_{j'}}{2} \right)^{2}$ where $j<j'$
- Interval metric scale: $\delta_{jj'}^{2}=\left( j-j' \right)^{2}$
- Ratio metric scale: $\delta_{jj'}^{2}=\left( \frac{j-j'}{j+j'} \right)^{2}$

[1] Fleiss JL, Cohen J, Everitt BS. Large sample standard errors of kappa and weighted kappa. Psychological Bulletin 1969, 72(5): 323-327.
[2] Fleiss JL, Levin B, Paik MC. Statistical methods for rates and proportions (3rd ed.). John Wiley & Sons, Inc. Hoboken, New Jersey; 2003.
[3] Krippendorff K. Estimating the reliability, systematic error, and random error of interval data. Educational and Psychological Measurement 1970, 30:61-70.
