# Supplementary material for: Measuring inter-rater reliability for nominal data – which coefficients and confidence intervals are appropriate?
Source: BMC Med Res Methodol. 2016 Aug 5;16:93. doi: 10.1186/s12874-016-0200-9 (PMC4974794; doi:10.1186/s12874-016-0200-9)
Supplement: Additional file 2: — Available software – implementation of Fleiss’ K and/or Krippendorff’s alpha in most common statistical software programs used in epidemiology/biometry. (DOCX 14 kb) [file 12874_2016_200_MOESM2_ESM.docx]

**Additional file 2: Available Software for Fleiss’ K and/or Krippendorff’s alpha**

In the following we will itemize for the most common statistical software programs used in epidemiology if Fleiss’ K and/or Krippendorff’s alpha is implemented.

In SAS (SAS Institute, Cary NC), Fleiss’ K is implemented in the FREQ procedure. For Krippendorff’s alpha there exists a macro for the calculation of the point estimate with confidence interval, implemented by Hayes [1]. However it should be noted that the bootstrap algorithm from Krippendorff is used there. Furthermore, the macro runs only in a SAS/IML environment.

For SPSS (IBM Corp., Armonk, NY), Fleiss’ K is implemented in the crosstabs menu. The asymptotic confidence interval can be calculated only by hand using the asymptotic standard error; furthermore, the standard bootstrap confidence interval can be calculated. For Krippendorff’s alpha a macro is implemented by Hayes analogous to the SAS-macro [1], again with Krippendorff’s bootstrap algorithm.

In R (R Core Team, Vienna, Austria) there is the package irr (version 0.84) from Gamer et al. [2], which calculates Fleiss’ K and Krippendorff’s alpha, but both without confidence intervals. There is a small error in the estimation of the coincidence matrix for Krippendorff’s alpha if there are no missing values. In the upcoming actualized version this error will be corrected (personal communication). An R-program for the calculation of Krippendorffs alpha with the standard bootstrap confidence interval as applied by us was written by Gruszczynski and can be downloaded via GitHub [3].

For Stata (StataCorp., College Station, TX), there exists also a macro, written by Klein, provided via the IDEAS homepage, which allows calculating point estimates and CIs for Krippendorff’s alpha (again Krippendorff’s bootstrap approach) [4]. Fleiss’ K is implemented in Stata’s kappa command; asymptotic CIs have to be calculated by hand using Fleiss’ standard error.

[1] Hayes A. http://www.afhayes.com/spss-sas-and-mplus-macros-and-code.html. Last access 2015/06/23.
[2] Gamer M, Lemon J, Singh P. Various coefficients of interrater reliability and agreement. Package ‘irr’, version 0.84; 2015.
[3] Gruszczynski M. https://github.com/MikeGruz/kripp.boot. Last access 2015/06/23.
[4] Klein D. Kalpha. Stata module to compute Krippendorff’s alpha-reliability. https://ideas.repec.org/c/boc/bocode/s457862.html. Last access 2015/07/29.
