# Supplementary material for: Measuring inter-rater reliability for nominal data – which coefficients and confidence intervals are appropriate?
Source: BMC Med Res Methodol. 2016 Aug 5;16:93. doi: 10.1186/s12874-016-0200-9 (PMC4974794; doi:10.1186/s12874-016-0200-9)
Supplement: Additional file 3: — R-script k_alpha – syntax, explanation, and analysis of a fictitious data set. (DOCX 25 kb) [file 12874_2016_200_MOESM3_ESM.docx]

**Additional file 3: R-script k_alpha – syntax, explanation, and analysis of a fictitious data set**

When reading in a dataset, observations need to be defined as rows and raters as columns, in contrast to the function kripp.alpha in the R-package irr. The R-script offers several options. First, the type-one error can be defined, which is pre-specified as 5%, and the number of bootstrap samples can be changed from the pre-defined value of 1000 (which were set in accordance with Efron [1]). Second, the measurement scale is pre-specified as nominal, but can be changed to ordinal, interval metric or ratio metric. Missing values are allowed; however, for Fleiss’ K all individuals with missing values will be excluded.

The output code includes the number of effectively used individuals, raters and categories, the observed agreement, the point estimates and the limits of the confidence intervals. If the measurement scale is not nominal, Fleiss’ K is not calculated and a note is printed.

*Syntax*

# input:

# ratings_t = data set

# alpha_q = two-sided type one error, default = 0.05

# nboot = number of Bootstrap samples, defaul=1000

# scaling = measurement scale ("nominal", "ordinal", "interval", "ratio"), default="nominal"

k_alpha=function(ratings_t, alpha_q=0.05, nboot=1000, scaling="nominal"){

# functions for Fleiss' K and Krippendorff's alpha

# function for the estimation of Fleiss' K

k_func=function(N,n,k,ratings,categ){

# n_ij = number of raters who classed subject i in category j

n_ij=matrix(ncol=k,nrow=N)

step=1

for (j in categ){

for (i in 1:N){

n_ij[i,step]=sum(as.numeric(ratings[i,]==j))

}

step=step+1

}

# estimation of K_j

p_j=apply(n_ij,2,sum)/(N*n)

q_j=1-p_j

k_j=1-apply(n_ij*(n-n_ij),2,sum)/(N*n*(n-1)*p_j*q_j)

# estimation of the overall K

k_t=sum(p_j*q_j*k_j)/sum(p_j*q_j)

return(list(k_t,p_j,q_j))

}

# Function for the estimation of alpha

alpha_func=function(k,n,N,ratings,categ){

# conicidence matrix

CM=matrix(ncol=k,nrow=k,0)

vn <- function(datavec) sum(!is.na(datavec))

if (any(is.na(ratings))) mc=apply(ratings, 1, vn) - 1 else mc=rep(n-1, N)

for (i in 1:N){

for (j in 1:(n-1)){

for (jt in (j+1):n){

if (!is.na(ratings[i,j]) && !is.na(ratings[i,jt])){

index1=which(categ == ratings[i,j])

index2=which(categ == ratings[i,jt])

CM[index1,index2]=CM[index1,index2]+(1+(index1==index2))/mc[i]

if (index1!=index2){

CM[index2,index1]=CM[index1,index2]

}

}

}

}

}

nmv <- sum(apply(CM, 2, sum))

nc=apply(CM,1,sum)

ncnk=matrix(0,nrow=k,ncol=k)

# matrix of expected disagreement

D_e=matrix(0,ncol=k,nrow=k)

for (C in 1:k) {

for (Ct in 1:k) {

if (C==Ct){

D_e[C,Ct]=nc[C]*(nc[Ct]-1)/(nmv-1)

}

if (C!=Ct){

D_e[C,Ct]=nc[C]*nc[Ct]/(nmv-1)

}

ncnk[C,Ct]=nc[C]*nc[Ct]

ncnk[Ct,C]=ncnk[C,Ct]

}

}

# matrix of metric differences

diff2=matrix(0,nrow=k,ncol=k)

# nominal

if (match(scaling[1], "nominal", 0)){

diff2=matrix(1,ncol=k,nrow=k)

diag(diff2)=0

}

# ordinal

if (match(scaling[1], "ordinal", 0)) {

for (C in 1:k){

for (Ct in 1:k){

if (C!=Ct){

tmp=nc[C:Ct]

diff2[C,Ct]=(sum(tmp)-nc[C]/2-nc[Ct]/2)^2

diff2[Ct,C]=diff2[C,Ct]

}

}

}

}

# interval

if (match(scaling[1], "interval", 0)){

for (C in 1:k){

for (Ct in 1:k){

if (C!=Ct){

diff2[C,Ct]=(as.numeric(categ)[C]-as.numeric(categ)[Ct])^2

diff2[Ct,C]=diff2[C,Ct]

}

}

}

}

# ratio

if (match(scaling[1], "ratio", 0)){

for (C in 1:k){

for (Ct in 1:k){

if (C!=Ct){

diff2[C,Ct]=((as.numeric(categ)[C]-as.numeric(categ)[Ct])/

(as.numeric(categ)[C]+as.numeric(categ)[Ct]))^2

diff2[Ct,C]=diff2[C,Ct]

}

}

}

}

# point estimator of Krippendorff's alpha

tmp=diff2*CM

num=sum(tmp)

tmp=diff2*D_e

den=sum(tmp)

if (den>0){

alpha_boot=1-num/den

}

if (den<=0){

alpha_est=NA

}

return(alpha_boot)

}

################################################

############ Fleiss' K #####################

################################################

# check, if measurement scale is nominal

if (match(scaling[1], "nominal", 0)){

# deleting all subjects with missing values

ratings_c <- as.matrix(na.omit(ratings_t))

# N = number of subjects, n = number of raters, k = number of categories

N_c=nrow(ratings_c)

v=function(dat){min(dat)==max(dat)}

agr_k=sum(apply(ratings_c,1,v))/N_c

# check, if there are at least two individuals without missing values

if (N_c<2){

print("There are less than two subjects withour missing values. Therefore, Fleiss' K cannot be calculated.")

}

if (N_c>=2){

n_c=ncol(ratings_c)

categ_c=levels(as.factor(ratings_c))

k_c=length(categ_c)

# point estimator of Fleiss` K

k_=k_func(N_c,n_c,k_c,ratings_c,categ_c)

k_est=k_[[1]]

p_j=k_[[2]]

q_j=k_[[3]]

########### asymptotic confidence interval ######################

# estimation of the standard error

se_k=(sqrt(2)/(sum(p_j*q_j)*sqrt(N_c*n_c*(n_c-1))))*sqrt(sum(p_j*q_j)^2-sum(p_j*q_j*(q_j-p_j)))

# asymptotic confidence interval for Fleiss' K

CI_asymp_k=k_est+c(-1,1)*qnorm(1-alpha_q/2)*se_k

}

}

#######################################################

############ Krippendorff's alpha #####################

#######################################################

# deleting all subject with less than two ratings

f=function(x)sum(!is.na(x))

# deleting all subjects with only one rating

ratings=as.matrix(ratings_t[apply(ratings_t,1,f)>1,])

# N = number of subjects, n = number of raters, k = number of categories

N_kr=nrow(ratings)

v=function(dat){min(dat,na.rm=TRUE)==max(dat,na.rm=TRUE)}

agr_alpha=sum(apply(ratings,1,v))/N_kr

n_kr=ncol(ratings)

categ=levels(as.factor(ratings))

k_kr=length(categ)

# point estimator of Krippendorff's alpha

alpha_est=alpha_func(k_kr,n_kr,N_kr,ratings,categ)

##################################################################

########## Bootstrap confidence intervals #########################

##################################################################

# K and alpha in each Bootstrap sample

k_boot=0

alpha_boot=0

for (iboot in 1:nboot){

if (match(scaling[1], "nominal", 0)){

index.new=sample(seq(1,N_c,1),N_c,replace=TRUE)

ratings_b=ratings_c[index.new,]

n=ncol(ratings_b)

categ=levels(as.factor(ratings_b))

k=length(categ)

k.b<-k_func(N_c,n,k,ratings_b,categ)[[1]]

k_boot=c(k_boot,k.b)

}

f=function(x)sum(!is.na(x))

# deleting all subjects with only one rating

index.new=sample(seq(1,N_kr,1),N_kr,replace=TRUE)

ratings_b=ratings[index.new,]

n=ncol(ratings)

categ=levels(as.factor(ratings))

k=length(categ)

alpha_b=alpha_func(k,n,N_kr,ratings_b,categ)

alpha_boot=c(alpha_boot,alpha_b)

}

# confidence interval using the percentiles from the Bootstrap samples

if (match(scaling[1], "nominal", 0)){

CI_boot_k=quantile(k_boot[-1],probs=c(alpha_q/2,1-alpha_q/2), na.rm=TRUE)

}

# confidence interval using the percentiles from the Bootstrap samples

CI_boot_alpha=quantile(alpha_boot[-1],probs=c(alpha_q/2,1-alpha_q/2), na.rm=TRUE)

##########################################

############# Output #####################

##########################################

print(paste("The measurement scale is ",scaling,"."))

print("###### Fleiss' K ######")

if (!match(scaling[1], "nominal", 0)){

print(paste("Fleiss' K cannot be calculated, because it is only appropriate for nominal data."))

}

if (match(scaling[1], "nominal", 0)){

print(paste("The observed agreement in all complete cases is ",round(agr_k*100,1),"%."))

print(paste("N (number of subjects without missing values) =", N_c))

print(paste("n (number of ratings) =", n_c))

print(paste("k (number of categories) = ", k_c))

print(paste("Point estimator of Fleiss' K =", round(k_est,4)))

print(paste("Asymptotic two-sided ", (1-alpha_q)*100,"% confidence interval for Fleiss' K:", round (CI_asymp_k[1],4)," ; ",min(1,round(CI_asymp_k[2],4))))

print(paste("Two-sided ", (1-alpha_q)*100,"% Bootstrap confidence interval for Fleiss' K:", round (CI_boot_k[1],4)," ; ",round(CI_boot_k[2],4)))

}

print("###### Krippendorff's alpha ######")

print(paste("The observed agreement in all cases with at least two ratings is ",round(agr_alpha*100,1),"%."))

print(paste("N (number of subjects with two or more ratings) =", N_kr))

print(paste("n (number of ratings) =", n_kr))

print(paste("k (number of categories) = ", k_kr))

print(paste("Point estimator of Krippendorff's alpha =", round(alpha_est,4)))

print(paste("Two-sided ", (1-alpha_q)*100,"% Bootstrap confidence interval for Krippendorff's alpha:", round(CI_boot_alpha[1],4)," ; ",round(CI_boot_alpha[2],4)))

if (match(scaling[1], "nominal", 0)){

return(invisible(list(obs.agr.k=agr_k,est.k=k_est,ci.asympt.k=CI_asymp_k,ci.boot.k=CI_boot_k,
 obs.agr.alpha=agr_alpha,est.alpha=alpha_est,ci.boot.alpha=CI_boot_alpha)))

}

if (!match(scaling[1], "nominal", 0)){

return(invisible(list(obs.agr.alpha=agr_alpha,est.alpha=alpha_est,ci.boot.alpha=CI_boot_alpha)))

}

}

# input:

# ratings_t = data set (rows = individuals, columns = raters), missing values coded by NA

# alpha_q = two-sided type one error, default = 0.05

# nboot = number of Bootstrap samples, default=1000

# scaling = measurement scale ("nominal", "ordinal", "interval", "ratio"), default="nominal"

# output:

# observed agreement for the complete cases and for all cases with at least two ratings (obs.agr.k, obs.agr.alpha)

# point estimators: est.k, est.alpha

# confidence intervals: ci.asympt_k, ci.boot,k, ci.boot.alpha

*# Fictitious data set*

ratings_t=matrix(ncol=3,nrow=10,c(5,5,5,3,5,5,1,4,4,3,3,3,4,4,5,1,3,4,3,3,3,1,1,3,2,2,5,3,3,4),byrow=T)

*# Default syntax*

test=k_alpha (ratings_t, alpha_q=0.05, nboot=1000, scaling="nominal")

"The measurement scale is nominal ."

"###### Fleiss' K ######"

"The observed agreement in all complete cases is 30 %."

"N (number of subjects without missing values) = 10"

"n (number of ratings) = 3"

"k (number of categories) = 5"

"Point estimator of Fleiss' K = 0.3323"

"Asymptotic two-sided 95 % confidence interval for Fleiss' K: 0.136 ; 0.5287"

"Two-sided 95 % Bootstrap confidence interval for Fleiss' K: 0.0476 ; 0.5636"

"###### Krippendorff's alpha ######"

"The observed agreement in all cases with at least two ratings is 30 %."

"N (number of subjects with two or more ratings) = 10"

"n (number of ratings) = 3"

"k (number of categories) = 5"

"Point estimator of Krippendorff's alpha = 0.3546"

"Two-sided 95 % Bootstrap confidence interval for Krippendorff's alpha: 0.0659 ; 0.5782"

*# Output components*

> test

$obs.agr.k

[1] 0.3

$est.k

[1] 0.3323442

$ci.asympt.k

[1] 0.1360078 0.5286806

$ci.boot.k

2.5% 97.5%

0.04758464 0.56363636

$obs.agr.alpha

[1] 0.3

$est.alpha

[1] 0.3545994

$ci.boot.alpha

2.5% 97.5%

0.06591505 0.57818182

[1] Efron B. Six questions raised by the bootstrap. Exploring the limits of bootstrap. Editors LePage R, Billard L. Technical Report No. 139. Division of Biostatistics, Stanford University. Wiley & Sons, New York; 1992.
