# Supplementary material for: Measuring inter-rater reliability for nominal data – which coefficients and confidence intervals are appropriate?
Source: BMC Med Res Methodol. 2016 Aug 5;16:93. doi: 10.1186/s12874-016-0200-9 (PMC4974794; doi:10.1186/s12874-016-0200-9)
Supplement: Additional file 4: — Case study – description, dataset, syntax, and output. (DOCX 21 kb) [file 12874_2016_200_MOESM4_ESM.docx]

**Additional file 4: Case study – description, dataset, syntax, and output**

*Description of the case study*

In order to illustrate the theoretical considerations learnt from the simulation study, we applied the same approach to a real world dataset focusing on the inter-rater agreement in the histopathological assessment of breast cancer as used for epidemiological studies and clinical decision-making. The first n=50 breast cancer biopsies of the year 2013 that had been sent in for routine histopathological diagnostics at the Institute of Pathology, Diagnostik Ernst von Bergmann GmbH (Potsdam, Germany), were retrospectively included in the study. For the present study, the samples were independently re-evaluated by four senior pathologists, who are experienced in breast cancer pathology and immunohistochemistry, and who were blinded to the primary diagnosis and immunohistochemical staining results. The lesions had primarily been identified as invasive ductal (n=46), invasive lobular (n=3) or mucinous (n=1) breast carcinoma; immunohistochemical examinations of estrogen receptor (ER), progesterone receptor (PR), proliferation index (MIB-1) and human epidermal growth factor receptor 2 (HER-2) had primarily been executed at the time of clinical diagnosis, using a standardized staining system (BenchMark GX, Roche, Berlin, Germany). Evaluation of immunohistochemistry had been carried out according to the German guidelines for breast cancer [1] (pp. 96-98). For ER and PR, the staining intensity was graded as absent (0), weak (1), moderate (2) or strong (3), the percentage of immunoreactive tumor cells was estimated in steps of ten-percent and pooled in groups as ‘no stained receptors’ (0), ‘< 10%’ (1), ‘10-50%’ (2), ‘51-80%’ (3) and ‘>80%’ (4). However, if the percentage was below ten percent, an attempt was made to assign percentages with accuracy of one percent-increments. Staining intensity and percentage were combined to a so-called immunoreactive score (IRS) [2]. For MIB-1 the percentage was evaluated in the same manner irrespective of the staining intensity. The HER-2 staining was evaluated according to current national and international practice [1 (pp. 99-100), 3]: Score 0, staining in no more than 10 % tumor cells; Score 1+, incomplete membranous staining in more than 10 % tumor cells; Score 2+, weak or moderate complete membranous staining in more than 10 % tumor cells; Score 3+, strong complete membranous staining in more than 10 % tumor cells. Additionally, we dichotomized the immunoreactive score (IRS) (which is used for ER detection and results from multiplication of ER staining intensity with the category of proportion of reactive cells): We regarded IRS 0-2 negative and IRS ≥3 positive for ER expression [2]. MIB-1 was defined as negative if less than 20% tumor cells were found, and above as positive [4]. HER-2 was negative if an original score of 0 or 1 was present, positive if it was 3, and intermediate if 2 [3]. We evaluated all measures of interest using Fleiss’ K and Krippendorff’s alpha with their CIs as described for the simulation study.

[1] Interdisciplinary S3 Guidelines for the Diagnosis, Treatment and Follow-up Care of Breast Cancer. Long version 3.0, Update 2012, German Cancer Society and German Society for Gynecology and Obstetrics

[2] Remmele W, Stegner HE. Recommendation for uniform definition of an immunoreactive score (IRS) for immunohistochemical estrogen receptor detection (ER-ICA) in breast cancer tissue. Pathologe 1987;8(3):138-40

[3] Carlson RW, Moench SJ, Hammond ME, Perez EA, Burstein HJ, Allred DC, Vogel CL, Goldstein LJ, Somlo G, Gradishar WJ, Hudis CA, Jahanzeb M, Stark A, Wolff AC, Press MF, Winer EP, Paik S, Ljung BM; NCCN HER2 Testing in Breast Cancer Task Force. HER2 testing in breast cancer: NCCN Task Force report and recommendations. Journal of National Comprehensive Cancer Network 2006 Jul;4 Suppl 3:S1-22

[4] Rocca A, Farolfi A, Maltoni R, Carretta E, Melegari E, Ferrario C, Cecconetto L, Sarti S, Schirone A, Fedeli A, Andreis D, Pietri E, Ibrahim T, Montalto E, Amadori D. Efficacy of endocrine therapy in relation to progesterone receptor and Ki67 expression in advanced breast cancer. Breast Cancer Research and Treatment 2015;152:57-65.

*Example dataset*

| Rater A | Rater B | Rater C | Rater D |
| --- | --- | --- | --- |
| 4 | 4 | 3 | 4 |
| 0 | 0 | 0 | 0 |
| 4 | 4 | 4 | 4 |
| 0 | 2 | 2 | 2 |
| 4 | 4 | 4 | 4 |
| 0 | 0 | 0 | 0 |
| 0 | 0 | 1 | 0 |
| 3 | 4 | 3 | 4 |
| 2 | 2 | 2 | 2 |
| 3 | 3 | 3 | 4 |
| 4 | 4 | 4 | 4 |
| 4 | 3 | 3 | 3 |
| 0 | 0 | 0 | 0 |
| 3 | 3 | 3 | 4 |
| 4 | 3 | 4 | 4 |
| 3 | 4 | 4 | 4 |
| 3 | 4 | 3 | 4 |
| 0 | 0 | 0 | 0 |
| 4 | 4 | 4 | 4 |
| 4 | 4 | 4 | 4 |
| 0 | 0 | 0 | 0 |
| 0 | 0 | 3 | 0 |
| 4 | 4 | 3 | 4 |
| 4 | 4 | 3 | 4 |
| 3 | 4 | 3 | 3 |
| 0 | 0 | 0 | 0 |
| 4 | 4 | 3 | 4 |
| 4 | 4 | 4 | 4 |
| 4 | 4 | 3 | 4 |
| 1 | 2 | 1 | 4 |
| 3 | 3 | 2 | 3 |
| 4 | 3 | 3 | 4 |
| 2 | 2 | 2 | 2 |
| 2 | 2 | 3 | 3 |
| 0 | 0 | 0 | 0 |
| 4 | 4 | 3 | 4 |
| 3 | 4 | 3 | 4 |
| 4 | 4 | 3 | 3 |
| 0 | 0 | 0 | 0 |
| 4 | 4 | 4 | 4 |
| 0 | 0 | 1 | 0 |
| 4 | 4 | 4 | 4 |
| 3 | 4 | 3 | 4 |
| 3 | 1 | 2 | 2 |
| 2 | 2 | 2 | 2 |
| 2 | 2 | 2 | 0 |
| 0 | 0 | 0 | 0 |
| 4 | 4 | 4 | 4 |
| 3 | 4 | 3 | 3 |
| 0 | 0 | 0 | 0 |

*Syntax for example dataset* (see Table S 1)

k_alpha (data)

k_alpha (data, scaling=”ordinal”)

*Output for example syntax*

k_alpha (data)

[1] "The measurement scale is nominal ."

[1] "###### Fleiss' K ######"

[1] "The observed agreement in all complete cases is 44 %."

[1] "N (number of subjects without missing values) = 50"

[1] "n (number of ratings) = 4"

[1] "k (number of categories) = 5"

[1] "Point estimator of Fleiss' K = 0.5625"

[1] "Asymptotic two-sided 95 % confidence interval for Fleiss' K: 0.4964 ; 0.6286"

[1] "Two-sided 95 % Bootstrap confidence interval for Fleiss' K: 0.4454 ; 0.6652"

[1] "###### Krippendorff's alpha ######"

[1] "The observed agreement in all cases with at least two ratings is 44 %."

[1] "N (number of subjects with two or more ratings) = 50"

[1] "n (number of ratings) = 4"

[1] "k (number of categories) = 5"

[1] "Point estimator of Krippendorff's alpha = 0.5647"

[1] "Two-sided 95 % Bootstrap confidence interval for Krippendorff's alpha: 0.4524 ; 0.6683"

>k_alpha (data, scaling=”ordinal”)

[1] "The measurement scale is ordinal ."

[1] "###### Fleiss' K ######"

[1] "Fleiss' K cannot be calculated, because it is only appropriate for nominal data."

[1] "###### Krippendorff's alpha ######"

[1] "The observed agreement in all cases with at least two ratings is 44 %."

[1] "N (number of subjects with two or more ratings) = 50"

[1] "n (number of ratings) = 4"

[1] "k (number of categories) = 5"

[1] "Point estimator of Krippendorff's alpha = 0.8343"

[1] "Two-sided 95 % Bootstrap confidence interval for Krippendorff's alpha: 0.7122 ; 0.9007"
