# Supplementary material for: Measuring inter-rater reliability for nominal data – which coefficients and confidence intervals are appropriate?
Source: BMC Med Res Methodol. 2016 Aug 5;16:93. doi: 10.1186/s12874-016-0200-9 (PMC4974794; doi:10.1186/s12874-016-0200-9)
Supplement: Additional file 5: — Figures A1 and A2 – scatter plot of the point estimates of Fleiss’ K versus Krippendorff’s alpha and empirical coverage probability of the asymptotic confidence interval for Fleiss’ K. (DOCX 92 kb) [file 12874_2016_200_MOESM5_ESM.docx]

**Additional file 5: Figure A1 and A2 – scatter plot of the point estimates of Fleiss’ K versus Krippendorff’s alpha and empirical coverage probability of the asymptotic confidence interval for Fleiss' K.**


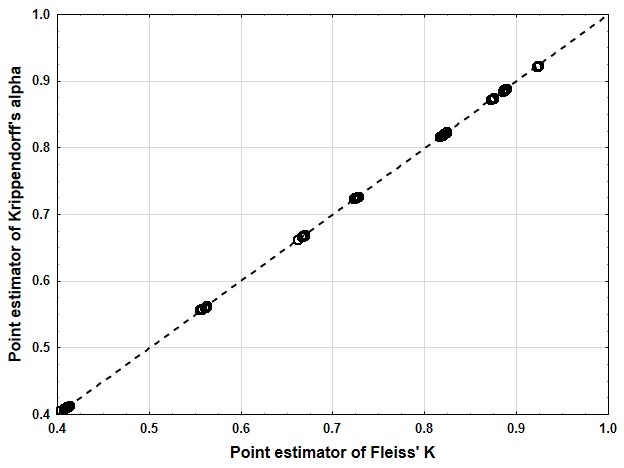


Figure A 1: Scatter plot of the point estimates (as mean over the 1000 simulation runs) of Fleiss’ K versus Krippendorff’s alpha. The dashed diagonal line represents equality.


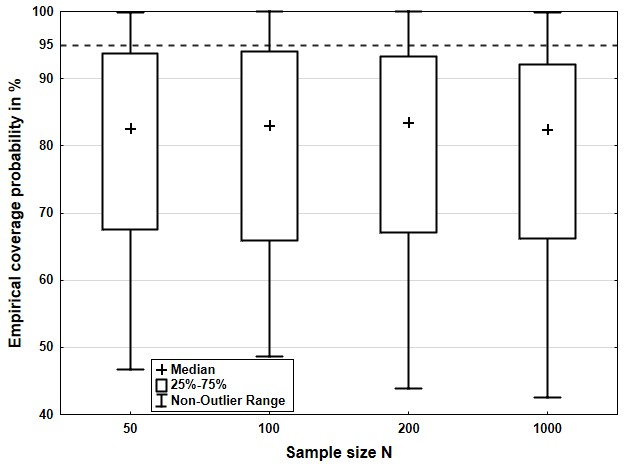


Figure A 2: Empirical coverage probability of the asymptotic confidence interval for Fleiss’ K with increasing sample size. The dotted line indicates the theoretical coverage probability of 95%.
